# Missouri Dental College

**Published:** 1867-08

**Authors:** 


### MISSOURI DENTAL COLLEGE.

It is with pleasure we announce the complete organization of the faculty of this Institution by the appointment of Dr. H. E. Peebles, to the chair of Operative Dentistry, and Dr———, to the chair of Mechanical Dentistry. This puts the institution in good regular working order. We presume no one will question for a moment the ability of these gentlemen to fill the positions assigned to them, in a satisfactory manner. This, combined with the zeal and energy which they are known to possess, in common with that of their able coadjutors, makes the success of this institution considerably more than problematical.

